# Effect of olfactory stimulation from aromatherapy on the autonomic nervous activity during aerobic exercises

**DOI:** 10.1038/s41598-024-61732-w

**Published:** 2024-05-16

**Authors:** Katsuki Okada, Koji Shimatani

**Affiliations:** 1 Ehime Prefectural Imabari Hospital, Imabari, Ehime 794-0006 Japan; 2 Prefectural University of Hiroshima, Mihara, Hiroshima 723-0053 Japan

**Keywords:** Cardiology, Health care, Medical research

## Abstract

Variations in the autonomic nervous system activity during exercise therapy in patients with cardiovascular diseases may lead to adverse events. Aromatherapy may reduce these adverse events by enhancing parasympathetic nervous activity (PNA). However, the effects of aromatherapy during exercise remain relatively unknown. This study aimed to evaluate the effect of aromatherapy on autonomic nervous activity during exercise and recovery. This randomized crossover study included 20 healthy men subjected to both aroma and placebo conditions which involved rest and moderate-intensity aerobic exercise on a cycle ergometer, followed by recovery. Blood pressure, heart rate variability indices, and SpO_2_ were measured during the rest, exercise, and recovery phases. Moreover, aroma preferences and emotional changes in response to the aroma were assessed. Under the placebo condition, high frequency (HF), root mean square of successive differences indices, and heart rate showed delayed recovery (*P* < 0.05). Furthermore, a moderate positive correlation was identified between aroma preference, pleasant emotions induced by aromatherapy, and the HF index (*P* < 0.05). These results indicate that aromatherapy facilitates the recovery of PNA after exercise. Furthermore, these effects were more pronounced among individuals who exhibited a stronger preference for and more positive emotions toward aromas.

## Introduction

Heart failure is a clinical syndrome in which various symptoms and signs caused by cardiac dysfunction result in decreased exercise capacity^1^. This condition is associated with high mortality rates and is anticipated to persistently rise^2^. Autonomic imbalance, such as increased sympathetic nervous activity (SNA) and decreased parasympathetic nervous activity (PNA), is a characteristic of heart failure and is associated with prognosis^3^. Involving in moderate-intensity aerobic exercises continuously for weeks to months improves autonomic imbalance in heart failure^4,5^. In contrast, SNA increased and PNA decreased during exercise^6–8^. This autonomic nervous system response triggers adverse events including excessive heart rate (HR)^9^, myocardial ischemia^9,10^, and ventricular arrhythmias^11,12^. In clinical practice, these adverse events hinder engaging in aerobic exercises of the appropriate intensity and duration. Furthermore, these effects may persist even after exercise. Yoga^13^, Taichi^14^, and deep breathing^7^ have demonstrated autonomic nervous system activity. However, combining these methods with exercise remains challenging. Aromatherapy is a complementary and alternative therapy that can be used during exercise. In particular, olfactory stimulation using a diffuser or a similar device can decrease HR^15^ and SNA^16,17^ and increase PNA^16,18–20^. Moreover, aromatherapy increases positive emotions^21^ and decreases negative emotions^17–19,22^. Furthermore, these effects have been reported in systematic reviews of cardiovascular disease^23^. However, most studies examining the effects of aromatherapy have been conducted at rest; nevertheless studies conducted during exercise are scarce. Therefore, this study aimed to examine the effects of aromatherapy during moderate-intensity aerobic exercise recommended for cardiac rehabilitation.

## Methods

### Participants

As previously mentioned, studies examining the effects of aromatherapy during exercise in patients with heart failure or in healthy individuals are lacking. Considering the possibility that aromatherapy may adversely affect exercise, we recruited healthy adult participants. Additionally, because the autonomic nervous system activity is influenced by the menstrual cycle^24,25^, only men were included. A total of 25 men were recruited through advertisements placed on the university campus. The exclusion criteria were anosmia, allergy to aroma, medical history affecting the autonomic nervous system, use of drugs or supplements that affect the autonomic nervous system, musculoskeletal diseases, and a history of smoking (previous/current smoker). These criteria are based on previous studies^18,19,26^. The experimental procedure was explained to the participants in paper form, and informed consent was obtained from all participants. This study was approved by the Ethics Committee of the Prefectural University of Hiroshima (approval no. 21MH023) and was conducted in accordance with the guidelines of the Declaration of Helsinki by the World Medical Association.

### Study design

This study was designed as a randomized crossover study, spanning three days with a washout period of more than one week but less than three weeks between days. The washout period was intended to allow for the persistence of aroma molecules in the olfactory epithelium and to account for exercise-related fatigue. During the initial laboratory visit, participants completed a questionnaire to assess their medical history, drug history, and fragrance allergies. Weight was measured using a digital scale (Digital Health Meter 1630, TANITA, Japan), and height was measured using a stadiometer (Stadiometer PA-200, UCHIDA, Japan). The International Physical Activity Questionnaire Short Version (IPAQ Short Ver.) was used to assess daily physical activity levels. Moreover, an olfactory function test and an aroma preference survey were conducted. Patients who did not meet the exclusion criteria underwent a cardiopulmonary exercise test (CPET). The main experiment commenced from the second laboratory visit. The researchers randomly categorized the participants into either the “aerobic exercise with aroma (aroma condition)” or the “aerobic exercise without aroma (placebo condition)” group using a computer-generated random sequence. On the third visit, aerobic exercise was performed with a crossover of conditions. The experimental period lasted 3 h, from 8:00 to 12:00, considering the effect of circadian rhythms on the autonomic nervous system^27,28^. To maintain laboratory standardization, the temperature and humidity of the room were set at 20–25 °C and 40–60%, respectively. Based on the criteria from previous studies^18,19,29–31^, the restrictions prior to the experiment were as follows: no excessive physical activity, alcohol, or nicotine for 24 h; no caffeine intake and only water allowed for oral intake from 21:00 the previous day; getting out of bed at least two hours before the experiment and consuming a light meal; and refraining from using perfume or other scented products on the day of the experiment.

### Olfactory function test

Based on previous studies^18,19^, an olfactory function test was conducted to confirm that none of the patients had anosmia. Three bottles were prepared in front of the participants: one containing lavender essential oil (Lavender France, TREE OF LIFE Co., Ltd., Japan), and the other two containing distilled water (Distilled water CAS RN:7732-18-5, Hayashi Pure Chemical Ind., Ltd., Japan). The participants were asked to select a bottle that was different from the others, and those who consistently selected the correct bottle three times were determined to not have anosmia.

### CPET

CPET was performed to determine the intensity for the main experiment. The participants exercised on a cycle ergometer (Corival ergometer cpet, KYOKKO BUSSAN Co., Ltd., Japan) using ramp protocols. In the ramp protocol, after maintaining a steady-state workload of 20 W for 4 min, the workload was increased continuously by 20 W per minute. Real-time measurements of oxygen uptake (VO_2_), carbon dioxide output (VCO_2_), tidal volume (TV), respiratory gas exchange ratio (RER), and respiratory rate (RR) were recorded using a breath-by-breath method during exercise with a pulmonary exercise stress monitoring system (Aeromonitor AE—300s, Minato Medical Science Co., Ltd., Japan). Additionally, HR was measured using an electrocardiogram (ECG) monitor (Bedside monitor BMS—3562, NIHON KOHDEN CORPORATION, Japan), blood pressure (BP) was measured using an automatic sphygmomanometer (Medical electronic blood pressure meter EBP—330, Minato Medical Science Co., Ltd., Japan), percutaneous oxygen saturation (SpO2) was measured using a pulse oximeter (Finger probe TL—201T, NIHON KOHDEN CORPORATION, Japan), and rate of perceived exertion (RPE) was assessed using the Borg scale. The termination criteria for CPET were as follows: Borg scale > 17 rotations per minute < 60, RER > 1.2, VO2 levering off, and reaching the target HR (220 – age × 0.85). After CPET, the aerobic threshold (AT) was determined using the V-slope method, and the peak VO2 was calculated using a dedicated software. Furthermore, %AT and %peak VO2 were calculated based on Japanese standard values.

### Aerobic exercise

**Figure 1:**
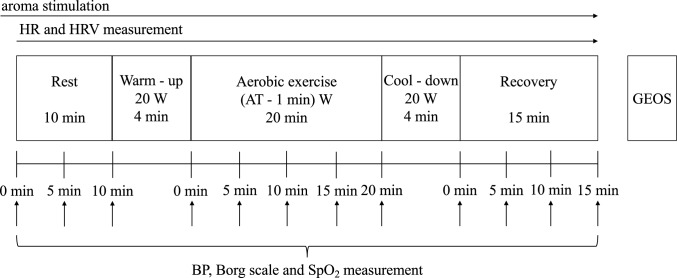
Experimental and data collection procedure. *HR* heart rate. *HRV* heart rate variability. *BP* blood pressure. *GEOS* geneva emotional and odor scale.

The experimental procedure is shown in Fig. 1. Aerobic exercise was designed in accordance with the cardiac rehabilitation guidelines in Japan^32^. After 10 min of rest in a sitting position, a 4 min warm-up was conducted using a cycle ergometer (Corival ergometer cpet, KYOKKO BUSSAN Co., Ltd., Japan) at 20 W. Subsequently, the participants engaged in 20 min of aerobic exercise at an intensity corresponding to Watts at AT-1 min (as determined during the CPET). The participants were instructed to maintain a cycling pace of 60–70 rpm. Following aerobic exercise, a 4 min cool-down period at 20 W was performed, followed by a 15 min recovery period in a sitting position. The participants were positioned in a resting posture, lightly touching the backrest, facing forward, and instructed not to fall asleep. Participants were instructed to breathe as usual. The transition to the next phase during the experiment was communicated to the participants through written instructions, considering the potential emotional changes due to the examiner’s verbal communication.

### Aroma stimulation

For aroma stimulation, essential oils were diffused in the room using an aroma diffuser (ESSWNTIAL OIL DIFFUSER aromore TL—EOD—A1, TREE OF LIFE Co., Ltd., Japan). Based on previous research on aromatherapy with essential oils^33^, the diffuser was positioned at a distance of approximately 1 m from the participant's nostrils, and the stimulation began just before the 10 min resting period in a sitting position before aerobic exercise. Lavender essential oil (Lavender France, TREE OF LIFE Co., Ltd., Japan) was used in the aroma condition, whereas distilled water (Distilled water CAS RN:7732-18-5, Hayashi Pure Chemical Ind., Ltd., Japan) was used in the placebo condition. The lavender essential oil was placed in the diffuser without dilution, following the manufacturer’s recommendations. The perceived intensity of aroma has been reported to decrease over time with prolonged exposure to the aroma. This suggests the potential occurrence of adaptation to the aroma^34,35^. Therefore, in reference to previous studies, we implemented a diffusion setting that alternates between 20 seconds of scent diffusion and 40 s of rest in order to address the possibility of rapid adaptation. Lavender is one of the most commonly used plants in aromatherapy for cardiovascular diseases, with no reported adverse effects^23^. Lavender oil possesses relaxation effects, reduces negative emotions^22^ and increases the PNA^19^. To account for the lingering presence of aroma molecules in the room, the aroma and placebo conditions were carried out on separate days, or the aroma condition followed the placebo condition. The participants were not informed about the presence or absence of an aroma or about the specific type of aroma.

### Outcomes

The data collection procedure is shown in Fig. 1. The primary outcomes were high frequency (HF), root mean square of successive differences (RMSSD), and standard deviation of R–R intervals (SDRR), which were examined using heart rate variability (HRV) analysis. Secondary outcomes were preference for aroma, HR, and emotional changes assessed using the Geneva Emotional and Odor Scale (GEOS) due to aroma, BP, Borg scale, SpO_2_ and low frequency (LF), and LF/HF examined using HRV analysis. HRV analysis was performed after all subjects had completed the experiment.

### Aroma preference survey

Preference for aroma may influence the autonomic nervous activity and cardiorespiratory blood pressure^36,37^. Therefore, an olfactory function test was conducted to investigate aroma preference. Preference was evaluated using the Visual Analog Scale (VAS), which ranged from 0 to 10 cm, with 0 indicating “dislike” and 10 indicating “like.”

### Emotional changes in response to aroma

Following the recovery period after aerobic exercise, emotional changes in response to the aroma were assessed using the GEOS^38^. The GEOS utilized a VAS graded from 0 to 10 cm, where 0 represented “not strong” and 10 represented “extremely strong” for evaluating emotional responses across five mood states: pleasant (good), unpleasant (bad, uncomfortable, disgusted, frustrated, and stressful), sensual (romantic), relaxed (relax, calm, drowsy), and refreshed (fresh, active).

### Heart rate and heart rate variability

The HR during the entire duration of aerobic exercise was continuously monitored using three-lead ECG electrodes. ECG data were used for HR and HRV analysis. HR and HRV indices were analyzed using HRV analysis software (Lab Chart 8, Nihon Bioresearch Inc., Japan) at the following time intervals: Rest (rest 5–10 min), Exercise (exercise 15–20 min), Rec1 (recovery period 0–5 min), Rec2 (recovery period 5–10 min), and Rec3 (recovery period 10–15 min). HRV analysis is a noninvasive method used to assess cardiovascular autonomic nervous activity by analyzing the continuous R–R interval^39,40^. Its reliability and validity have been previously reported^41,42^. Both digital and manual filtering were applied to eliminate artifacts. The recorded data included at least 256 consecutive RR intervals, with more than 95% exhibiting sinus HR. In this study, both frequency and time domain analyses were conducted. The frequency domain included very low frequency (VLF = 0–0.04 Hz), LF (0.04–0.15 Hz), HF (0.15–0.45 Hz), and LF/HF ratios. The time domain involved the RMSSD and SDRR.

### Blood pressure, percutaneous oxygen saturation and rate of perceived exertion

BP, SpO_2_, and RPE during aerobic exercise were measured at the end of each phase or every five minutes using an automatic sphygmomanometer (Medical Electronic Blood Pressure Meter EBP—330, Minato Medical Science Co., Ltd., Japan), a pulse oximeter (Wristwatch pulse oximeter PULSOX—300, KONICA MINOLTA, INC., Japan), and a Borg scale^43^. The Borg scale was presented to the participants on paper, and they indicated their responses by pointing. We conducted analyses using the collected data at specific time points: Rest (rest 10 min), Exercise (exercise 20 min), Rec1 (recovery period 5 min), Rec2 (recovery period 10 min), and Rec3 (recovery period 15 min).

### Statistical analyses

The Gaussian distribution of the data was assessed using the Shapiro–Wilk normality test. Descriptive statistics were used to characterize the sample, presenting parametric distributions as means (standard deviations) and non-parametric distributions as medians (interquartile ranges). To analyze the emotional changes in response to aroma using GEOS between the aroma and placebo conditions, parametric distributions were assessed using a paired t-test, whereas nonparametric distributions were assessed using the Wilcoxon signed-rank sum test. To examine the influence of aroma inhalation on blood pressure, percutaneous oxygen saturation, rate of perceived exertion, HR and HRV indices, a two-way repeated measures analysis of variance (ANOVA) was conducted on parametric distributions to assess the effects of aroma, time, and their interaction. Additionally, Friedman tests were performed for nonparametric distributions. If a main effect of time was observed, a one-way ANOVA with repeated measures was performed. When an interaction between aroma and time was identified, paired t-tests were performed at each time point. Mauchly’s test was used to check for sphericity violations when conducting ANOVA with repeated measures. The Greenhouse–Geisser correction was applied when sphericity was violated. Bonferroni's multiple comparisons were used for post-hoc tests. Basal heart rate, autonomic nervous activity, and BP differ among individuals. Therefore, these indices at rest were standardized to zero, and their changes were compared. The correlation between aroma preference and HRV indices was assessed using Pearson’s product-moment correlation coefficient for parametric distributions and Spearman’s rank correlation coefficient for nonparametric distributions. Similarly, the correlation between emotional changes using the GEOS and HRV indices was analyzed. Statistical significance was set at 5% for all analyses. All statistical analyses were performed using EZR (Saitama Medical Center, Jichi Medical University, Saitama, Japan)^44^, which is a graphical user interface for R (R Foundation for Statistical Computing, Vienna, Austria, version 3.4.1). More precisely, it is a modified version of the R Commander (version 2.4-0) that was designed to add statistical functions frequently used in biostatistics.

## Results

**Figure 2:**
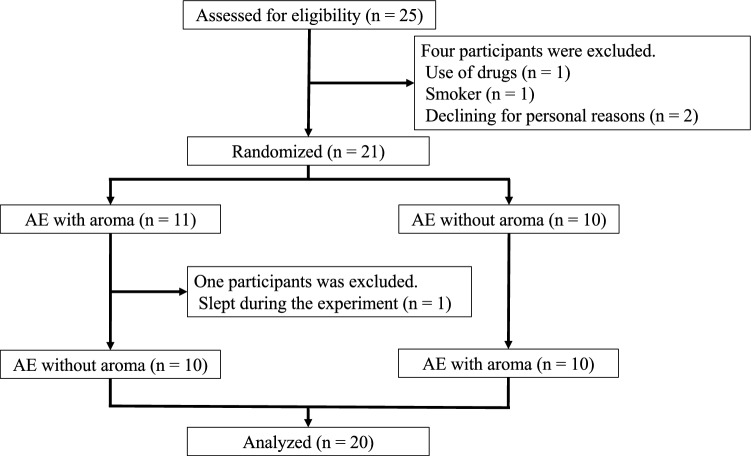
Flow diagram of the progress through the phases of a crossover study. *AE* aerobic exercise.

A flow diagram of the trial course for all participants is shown in Fig. 2. The study initially included 25 participants, and only 20 participants were included in the final analysis. The baseline characteristics of the study participants are presented in Table 1. We obtained comments from all participants indicating the perception of the aroma during the exercise. We believe that the intermittent aroma diffusion strategy helped avoid adaptation.

**Table 1:** Participant characteristics.

Variables	Mean (SD) or median (IQR)	Range
Age (years)	23 (21.0–24.5)	[20–36]
Body composition		
Height (m)	171.60 (6.63)	[160.1–180.2]
Weight (kg)	65.23 (10.84)	[46.6–86.0]
BMI (kg/m^2^)	22.06 (10.84)	[18.00–28.00]
CPET		
RER	1.12 (0.06)	[0.99–1.12]
AT (V-slope mL/min/kg)	15.50 (3.33)	[9.60–20.40]
%AT (%)	85.35 (18.38)	[52.17–110.87]
Peak VO2 (mL/min/kg)	25.38 (5.11)	[15.10–32.90]
%peak VO2 (%)	76.64 (15.08)	[45.07–98.21]
Peak work rate (W)	163.70 (28.83)	[106.60–216.00]
% peak work rate (%)	76.20 (11.50)	[51.07–93.19]
IPAQ short form		
Total physical activity (MET-min/week)	2925 (940–4782)	[0–8798]
Physical activity level	n (%)	
Low	4 (20)	
Moderate	7 (35)	
High	9 (45)	
Aroma preference survey		
Aroma preference	6.11 (2.05)	[1.2–9.4]

Parametric and nonparametric distributions are presented as mean (standard deviation) and median (inter quartile range), respectively, with minimum and maximum values shown.

*BMI* body mass index, *CPET* cardiopulmonary exercise test, *RER* respiratory gas exchange ratio, *AT* aerobic threshold, *VO2* oxygen uptake, *IPAQ* international physical activity questionnaire, *MET* metabolic equivalent.

### Emotional changes in response to aroma

Table 2 shows the emotional changes in response to aroma immediately after the recovery period following aerobic exercise, as assessed using the GEOS. The pleasant (*P* < 0.001), relaxed (*P* < 0.001), refreshing (*P* < 0.001), and sensual (*P* = 0.028) emotions were significantly higher in the aroma condition than in the placebo condition. No significant differences in the emotion of displeasure were observed between the aroma and placebo groups (*P* = 0.57).

**Table 2:** Emotional changes in response to aroma immediately after the recovery period during aerobic exercise by GEOS.

	Aroma	Control	Probability
Emotion			
Pleasure	6.22 (2.21)	2.80 (2.25)	< 0.001
Displeasure	1.52 (0.20–2.13)	1.38 (0.10–1.58)	0.57
Relax	6.97 (1.40)	3.22 (2.36)	< 0.001
Refresh	5.72 (2.36)	2.54 (2.50)	< 0.001
Sensual	2.40 (2.60)	1.06 (1.69)	0.028

Parametric distributions were analyzed with the paired t-test, and nonparametric distributions were analyzed with the Wilcoxon signed rank sum test, presented as mean (standard deviation) or median (inter quartile range), respectively.

### Heart rate and heart rate variability

**Figure 3:**
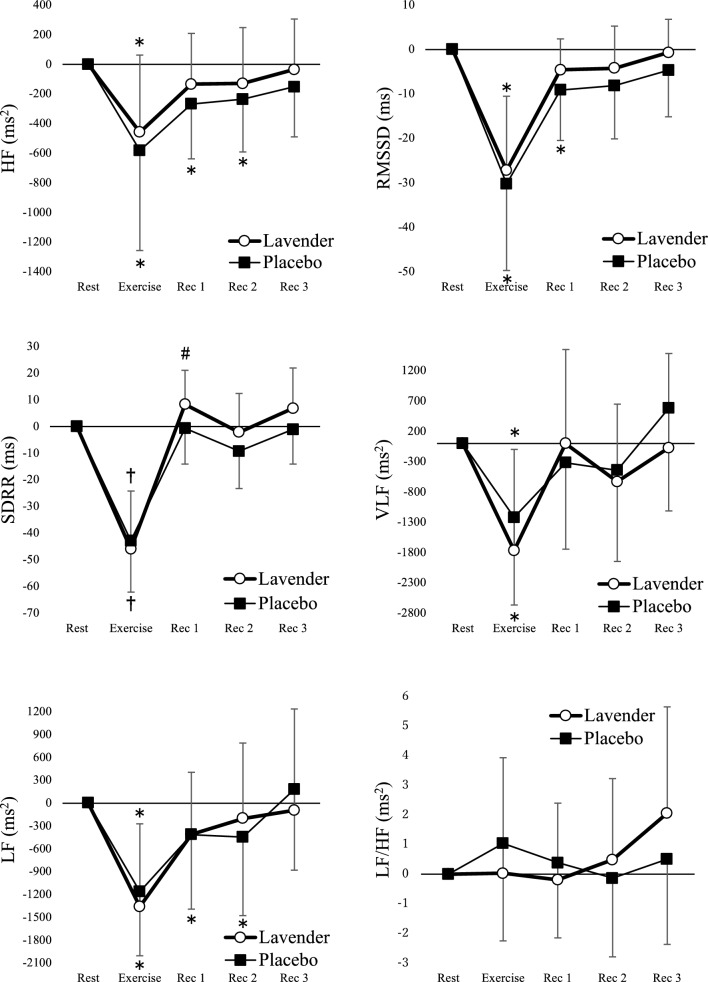
Mean values and their respective standard deviations of indices in the HRV analysis during rest, exercise, and recovery (Rec), obtained from the aroma and control conditions. These indices at rest were standardized to zero, and their changes were compared. *Values with significant differences than at rest (Friedman test followed by the Bonferroni correction; *P* < 0.05); †Values with significant differences than at rest (One way ANOVA for repeated measures followed by the Bonferroni correction; *P* < 0.05); #Values with significant difference than during aroma and control conditions (paired t-test; *P* = 0.028).

The behavior of the frequency and time domain data in the HRV analysis under the aroma and placebo conditions compared with the resting state is shown in Fig. 3. We observed a significant effect of time on the HF, RMSSD, VLF, and LF indices (aroma condition, *P* < 0.001; placebo condition, *P* < 0.001); however, there was no significant effect of time on the LF/HF index (aroma condition, *P* = 0.15; placebo condition, *P* = 0.44). HF, RMSSD, and LF indices were significantly lower only during exercise in the aroma condition than in the rest state. Conversely, in the placebo condition, the HF and LF indices were significantly lower until Rec2, and the RMSSD index was significantly lower until Rec1. The VLF index decreased significantly only during exercise under both conditions.

Regarding the SDRR index, a significant effect of time was observed (*P* < 0.001), whereas the interaction between aroma and time was almost significant (*P* = 0.073). Subsequent statistical analyses revealed that the SDRR index was significantly higher for Rec1 in the aroma condition than in the placebo condition (*P* = 0.028). No statistically significant differences were observed between Rec2 (*P* = 0.12) and Rec3 (*P* = 0.10). We observed no effect of time on LF/HF in both aroma (*P* = 0.15) and placebo (*P* = 0.44) conditions.

**Figure 4:**
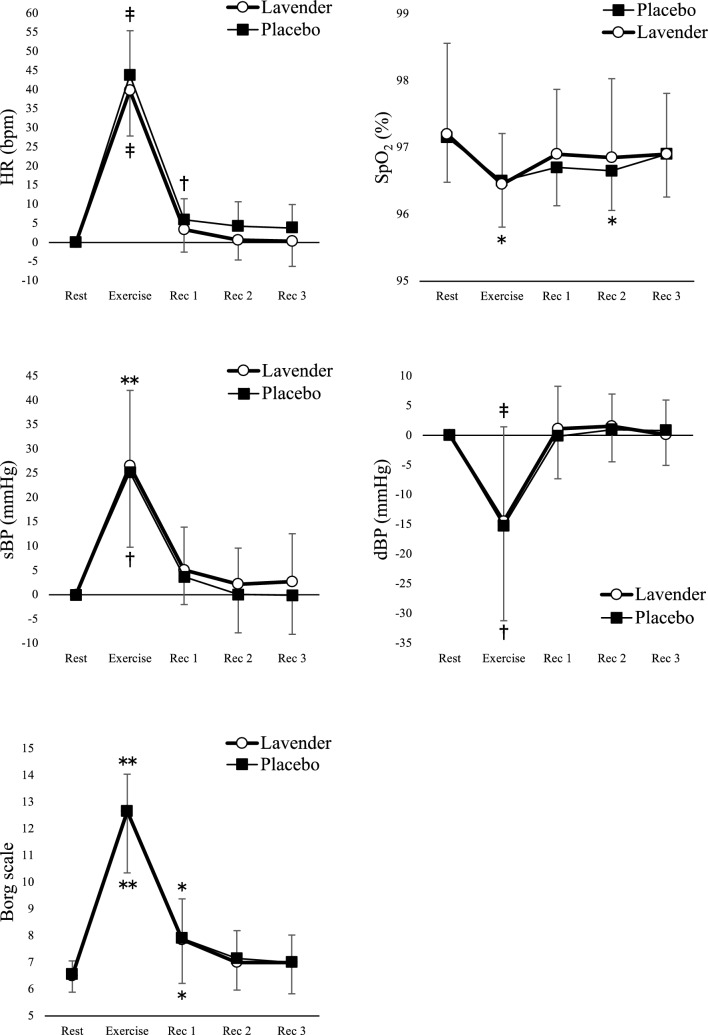
Mean values and their respective standard deviations of indices in the hemodynamic parameters and RPE during rest, exercise, and recovery (Rec), obtained from the aroma and control conditions. HR and BP at rest were standardized to zero, and their changes were compared. A significant difference by Friedman test followed by the Bonferroni correction was apparent than during at rest (* < 0.05, ** < 0.001); A significant difference by One way ANOVA for repeated measures followed by the Bonferroni correction was apparent than during at rest († < 0.05, ‡ < 0.001).

We observed a significant effect of time (*P* < 0.001) and no significant interaction between aroma and time (*P* = 0.45) on HR. HR was significantly higher only during exercise in the aroma condition than in the rest state. Conversely, in the control condition, HR was significantly higher until Rec1 (Fig. 4).

In the aroma condition, significant correlations were observed between preference and HF index during exercise, as well as between pleasant emotions and HF index during exercise (Table 3). However, no significant correlations were found between the other emotions and any of the HRV parameters during exercise (Table 3).

**Table 3:** Correlation between preference and emotions in aroma condition and HRV during exercise.

	HF		RMSSD		SDRR	
	Correlation coefficient	Probability	Correlation coefficient	Probability	Correlation coefficient	Probability
Preference	0.57	0.008	0.4	0.084	0.29	0.21
Pleasure	0.48	0.035	0.27	0.25	0.36	0.12
Unpleasant	− 0.13	0.59	0.029	0.90	0.19	0.42
Sensual	− 0.14	0.55	− 0.14	0.57	− 0.14	0.55
Relaxed	0.36	0.12	0.11	0.64	0.16	0.50
Refreshed	− 0.15	0.53	0.042	0.86	− 0.098	0.68

### Blood pressure, percutaneous oxygen saturation and rate of perceived exertion

The behavior of the BP, SpO_2_ and RPE in the aroma and placebo conditions compared to the resting state is shown in Fig. 4.

We observed a significant effect of time on both systolic BP (sBP) and diastolic BP (dBP) (*P* < 0.001). Compared to the rest condition, sBP was significantly higher during exercise in both conditions, whereas dBP was significantly lower during exercise.

For SpO_2_, a significant effect of time was observed (*P* < 0.001). Compared with the rest state, no significant differences were observed at any time for the SpO_2_ in the aroma condition. However, in the control condition, SpO_2_ was significantly lower during exercise and Rec2.

Regarding the rate of perceived exertion, we observed a significant effect of time on the Borg scale (*P* < 0.001). The Borg scale score was significantly higher for exercise and Rec1 under both conditions than in the rest condition.

## Discussion

To our knowledge, our study is the first to evaluate the influence of aromatherapy on the ANS during and recovery from moderate-intensity aerobic exercise recommended for cardiac rehabilitation. Our findings showed that aromatherapy (a) enhanced positive emotions as evaluated by the GEOS, (b) accelerated the recovery of PNA as evaluated by HRV analysis, and (c) accelerated the recovery of HR. However, aromatherapy did not influence SpO_2_, BP, or RPE, as evaluated using the Borg scale.

Increased physical activity and duration induce negative emotions^45,46^. Conversely, aromatherapy induces positive emotions^21^. In our study, aromatherapy increased positive emotions, even during moderate-intensity aerobic exercise. These results suggest that aromatherapy has the potential to counteract the negative emotions induced by exercise. The brain regions responsible for olfaction and emotion are closely interconnected anatomically, encompassing areas such as the amygdala, anterior cingulate cortex, insula, and orbitofrontal cortex^47^. We consider that aromatherapy activated these brain regions to induce positive emotions even during exercise.

In our study, we observed an accelerated recovery of HF, RMSSD, and HR under aromatic conditions. A positive correlation was also identified between aroma preference, pleasant emotions, and HF indices during exercise under the aroma condition. On the other hand, BP responses did not differ between the aroma and placebo conditions. HF and RMSSD, as analyzed using HRV, reflect the PNA^48–50^. Additionally, SDRR is a parameter of the overall HRV influenced by both SNA and PNA. SDRR represents the activation status of the autonomic nervous activity^51^.Taken together, these results suggest that aromatherapy accelerates the recovery of PNA, increases overall HRV, and further accelerates HR recovery after exercise. The results may also indicate that participants with a stronger preference for pleasant emotions to aromatherapy experience increased PNA during exercise. However, aromatherapy did not show any effect on BP during exercise and Rec conditions.

Aromatherapy in a seated position, combined with quiet and comfortable relaxation enhances PNA^16,18–20^, which could also decrease HR^15,52^. The findings of our study align with those of previous studies that have explored the effectiveness of aromatherapy in restful positions. Our study highlights the enduring efficacy of aromatherapy after moderate-intensity aerobic exercise, even during the recovery phase. In addition, the positive emotions induced by aromatherapy at rest have been linked to its positive effects on the autonomic nervous system^53,54^. Similar results were obtained in this study, even during exercise, as in previous studies. Previous studies have reported mixed results regarding the effect of aromatherapy on BP. Some studies have suggested a decrease in both sBP and dBP^55,56^, whereas others have found a reduction in sBP without significant changes in dBP^22,57^. Consequently, a definitive consensus on the effects of aromatherapy remains elusive. In our study, no significant differences were observed between the aroma and placebo conditions during either the exercise or the recovery phases. Accordingly, our findings diverge from those of previous studies that investigated the effects of aromatherapy on BP in restful positions.

Three mechanisms mediate the autonomic cardiovascular adjustments necessary for exercise performance: arterial baroreflex, central command, and skeletal muscle exercise pressor reflex. Upon activation, these mechanisms stimulate autonomic cardiovascular control centers in the brainstem, leading to increased SNA and decreased PNA. Consequently, HR, cardiac output, and cardiac contractility increase in direct proportion to the intensity of the exercise^58–60^. Additionally, during dynamic exercises such as cycling bicycle ergometers, sBP increases owing to heightened SNA. Conversely, dBP remains relatively stable or decreases slightly owing to reductions in total peripheral vascular resistance, which is attributed to muscle contraction metabolites and endothelium-derived relaxing factors. However, it is well established that olfactory stimuli influence emotions and physiological functions. Extensive research has explored the relationship between olfaction and the autonomic nervous system. Olfaction is mediated by olfactory neurons within the olfactory bulb, and this information is subsequently relayed to olfactory regions in the brain. Olfactory nerves transmit inputs to primary olfactory regions, such as the amygdala, piriform cortex, and entorhinal cortex, where olfactory information reaches secondary olfactory regions, including the hippocampus, thalamus, insula, and orbitofrontal cortex. Aromatherapy activates these brain regions, thereby modulating emotions and autonomic nervous activity^47,61^. Therefore, aromatherapy may increase PNA and decrease HR through a pathway distinct from the autonomic nervous changes induced by exercise. However, despite promoting PNA recovery during the post-exercise phase in our study, aromatherapy did not significantly affect BP. These results suggest that aromatherapy might counteract autonomic nervous system responses arising from exercise-induced changes, thereby fostering PNA recovery through a pathway. Nevertheless, aromatherapy may not possess the ability to counteract changes in BP. Furthermore, the current study indicates that the greater the preference for aroma and the more pleasant the emotions induced by aromatherapy, the stronger the effect of aromatherapy.

After lavender inhalation, no significant change in SpO_2_ was observed in previous studies^56,62^. In our study, although a statistically significant difference was observed under the placebo condition, the magnitude of this difference was minimal, and all participants maintained adequate SpO_2_ levels. Consequently, this result suggests that the effect of aromatherapy on SpO_2_ was not significant.

Regarding the RPE measured using the Borg scale, there was no difference between the two conditions. Previous studies have established a strong relationship between Borg scale scores and exercise intensity, as assessed by parameters such as HR and blood lactate levels. Therefore, it is an affordable, practical, and valid tool for monitoring and prescribing exercise intensity^39,63,64^. In our study, we observed an increase in the Borg scale score in tandem with an increase in HR. Under aroma conditions, the presence of heightened pleasant and relaxed emotions, as measured by the GEOS, corresponded to enhanced recovery of PNA and concomitant HR adjustments. Based on these findings, it was predicted that the RPE would decrease. However, the lack of a decrease in RPE could potentially be attributed to the relatively limited recovery of the HR observed. In general, 10 times the Borg scale is considered the predictive value of HR, and for every 10 beats decrease in HR, the Borg scale score is considered to decrease by one. Considering that the difference in HR between the aroma and placebo conditions in our study ranged from two to four beats, it can be deduced that this variance had minimal impact on the Borg scale.

As a novelty of our study, we highlight the use of moderate-intensity aerobic exercise recommended for cardiac rehabilitation instead of predominantly restful positions, which have been used in previous studies investigating the effect of aromatherapy on autonomic nervous activity. In previous studies, patients with cardiovascular disease underwent a symptom-limited exercise test, suggesting that increased SNA and HR induced myocardial ischemia, which persisted even after the end of exercise^9,10^. Ventricular arrhythmias are induced by increased SNA in a canine model of cardiovascular disease^11,12^. The results of this study suggest that aromatherapy can be used for the risk management of adverse events during moderate-intensity aerobic exercise recommended for cardiac rehabilitation.

Despite our efforts to control for variables that influenced the outcomes, our study had certain limitations that warrant consideration. First, the participants included in this study comprised healthy, relatively young men, and individuals using medications that affect the autonomic nervous system were deliberately excluded from participation. Therefore, the generalizability of our findings to women, older adults, and individuals with autonomic nervous system disorders remains unclear. Furthermore, while no adverse events, such as arrhythmias or myocardial ischemia, were encountered during either the exercise or recovery phases, it should be noted that although aromatherapy appeared to enhance autonomic nervous system recovery, it has not been definitively established whether aromatherapy has the capacity to suppress adverse events. Second, it is important to acknowledge that HRV analysis does not directly assess the autonomic nervous system, and there is no index to assess SNA alone^65,66^. Consequently, changes in the SNA were not recorded in this study. Nonetheless, HRV analysis has emerged as a simple and non-invasive measure of autonomic nervous system activity and is a promising quantitative marker of autonomic balance^41,67^. Moreover, the HRV analysis was influenced by respiratory variations. Although the HF index fails to exhibit identical fluctuations between spontaneous and controlled breathing, the RMSSD index can be employed regardless of breathing pattern^68,69^. Few studies indicated no significant changes in respiratory rates before and after lavender administration^55,56,70^. To minimize the impact of respiratory changes, the participants were instructed to maintain normal breathing during the rest and recovery phases. Considering these findings, we contend that respiratory changes are unlikely to have a significant impact on HRV. In addition, a previous study has reported that HRV indices reach a minimum during moderate to high-intensity exercise and do not change substantially thereafter^71^. In this study, we employed moderate-intensity exercise, and there is a possibility that HRV during the exercise phase may not be accurately assessed. Therefore, the results of the HRV analysis in this study are relevant for assessing changes during the recovery phase rather than during the exercise phase. Finally, there is a possibility that participants might not have been fully blinded to the aroma or placebo conditions. To mitigate placebo effects, a randomized crossover trial was conducted, wherein the sequence of conditions was concealed from the participants in advance. Nonetheless, we must acknowledge that the participants might have discerned differentiation during aroma inhalation. Consideration of the adverse events stemming from aromatherapy is also important. Although no adverse events have been reported in studies involving aromatherapy at rest for cardiovascular disease^23,56,57,72–74^, the safety of aromatherapy in individuals with cardiovascular disease during exercise remains unknown.

In conclusion, our study showed that aromatherapy elicits positive emotions during moderate-intensity aerobic exercise, a modality recommended for cardiac rehabilitation, and facilitates the recovery of PNA and HR after exercise. Future studies should explore whether the observed effects of aromatherapy during exercise can be extended to diverse populations, including patients with cardiovascular disease, and whether the effects of aromatherapy on the autonomic nervous system during exercise contribute to the reduction of adverse events.

## Data Availability

The data that support the findings of this study are available from the corresponding author. Contact K. Shimatani (shimatani@pu-hiroshima.ac.jp) for additional data and other materials.
